# Structural basis of small molecule ATPase inhibition of a human mitotic kinesin motor protein

**DOI:** 10.1038/s41598-017-14754-6

**Published:** 2017-11-09

**Authors:** Hee-Won Park, Zhujun Ma, Haizhong Zhu, Shimin Jiang, Robert C. Robinson, Sharyn A. Endow

**Affiliations:** 1 Structural Genomics Consortium, University of Toronto, Toronto, ON M5G 1L7, Canada; 2 Neuroscience & Behavioural Disorders Programme, Duke-National University of Singapore Medical School, Singapore, 169857 Singapore; 3 Cancer & Stem Cell Biology Programme, Duke-National University of Singapore Medical School, Singapore, 169857 Singapore; 4 Institute of Molecular and Cell Biology, A*STAR (Agency for Science, Technology and Research), Biopolis, Singapore, 138673 Singapore; 5 Department of Biochemistry, National University of Singapore, Singapore, 117597 Singapore; 6 Research Institute for Interdisciplinary Science, Okayama University, Okayama, 700-8530 Japan; 7 Department of Cell Biology, Duke University Medical Center, Durham, NC 27710 USA; 8 Present Address: Department of Biochemistry and Molecular Biology, Tulane School of Medicine, New Orleans, LA 70112 USA; 9 Present Address: AbbVie, Inc., North Chicago, IL 60064 USA; 10 Present Address: NTU Institute of Structural Biology, Nanyang Technological University, Singapore, 636921 Singapore

## Abstract

Kinesin microtubule motor proteins play essential roles in division, including attaching chromosomes to spindles and crosslinking microtubules for spindle assembly. Human kinesin-14 KIFC1 is unique in that cancer cells with amplified centrosomes are dependent on the motor for viable division because of its ability to cluster centrosomes and form bipolar spindles, but it is not required for division in almost all normal cells. Screens for small molecule inhibitors of KIFC1 have yielded several candidates for further development, but obtaining structural data to determine their sites of binding has been difficult. Here we compare a previously unreported KIFC1 crystal structure with new structures of two closely related kinesin-14 proteins, Ncd and KIFC3, to determine the potential binding site of a known KIFC1 ATPase inhibitor, AZ82. We analyze the previously identified kinesin inhibitor binding sites and identify features of AZ82 that favor binding to one of the sites, the α4/α6 site. This selectivity can be explained by unique structural features of the KIFC1 α4/α6 binding site. These features may help improve the drug-like properties of AZ82 and other specific KIFC1 inhibitors.

## Introduction

Kinesin motor proteins hydrolyze ATP to produce force and do work in cells. Among the 14 known groups in the kinesin family, at least seven perform roles in division, rather than vesicle/organelle transport, another major kinesin function^1^. The mitotic kinesins attach chromosomes to spindle fibers^2,3^ and mediate chromosome congression to the metaphase plate^4^ – they also crosslink and slide microtubules to assemble and elongate spindles, and destabilize microtubules, contributing to spindle dynamics and microtubule length regulation in the spindle^5–7^. Considerable interest has focused on these kinesins because of their diverse roles in division and the insights they provide into fundamental mechanisms of division. In addition, their study has produced new information about the mechanism by which the motors function. Despite their diverse functions, the kinesin proteins contain a common motor domain with highly conserved or invariant sequence motifs that mediate basic motor properties, such as microtubule binding and ATP hydrolysis. These motifs form a molecular signature of the kinesins – single amino acid changes in these motifs alter basic motor functions and create new phenotypes, revealing key features of the motor mechanism of function^8–11^.

Kinesin proteins show differences in motility that are characteristic of their group. For example, kinesin-14 motors move on microtubules towards the minus end instead of the plus end^12,13^. Notwithstanding their reversed directionality, kinesin-14 motors bind to the same site on microtubules^14^ and contain the same invariant sequence motifs as other kinesins^15^. Crystal structures show that the kinesin motor domain is highly conserved^16,17^, despite basic differences among kinesins in directionality and processivity, as well as force generation^8,18^.

Their essential roles in mitosis raise the possibility that targeting specific kinesins could inhibit or block the unregulated division associated with cancers, providing new targets for treatment. However, the roles of the motors in division represent a double-edged sword, since small molecules that inhibit the proteins produce detrimental effects in normal cells, as well as those that divide abnormally. These unwanted effects have raised concerns about strategies targeting kinesins to develop new cancer therapies.

An apparent exception exists for human kinesin-14 KIFC1, also known as HSET or CHO2 (hereafter referred to as KIFC1). KIFC1 is one of three kinesin-14 proteins in humans, together with KIFC2 and KIFC3, and is expressed at low levels in almost all adult tissues except testis, where its expression levels are high. Reduced KIFC1 expression results in a rare male infertility disease characterized by defective acrosome formation and failure to elongate sperm heads^19^. In contrast to its low expression in other cells, KIFC1 shows high expression in many cancer cells^20^. Depletion of KIFC1 in these cells causes the formation of multipolar spindles, reducing cell viability. In normal cells, KIFC1 has been shown to bind to a centrosomal protein^21^ – in cancer cells with amplified centrosomes, it binds to and clusters centrosomes to promote bipolar spindle formation, preventing formation of multipolar spindles and cell death.

Because of its elevated expression in different cancer cells and the demonstrated dependence on KIFC1 for viability of these cells, together with the relative insensitivity of normal cells to its depletion, KIFC1 has been the target of several small molecule inhibitor screens. The screens have resulted in the identification of three compounds, CW079^22^, SR31527^23^ and AZ82^24^. The most potent of the three compounds, AZ82, specifically inhibits KIFC1 with a *K*_*i*_ of 0.043 μM^24^. Extensive biochemical assays have shown that AZ82 has little effect on KIFC1 without microtubules, but it is reported to inhibit both ATP binding to the KIFC1-microtubule (MT) complex and dissociation of ATP/ADP from the complex^24^. Despite the thorough kinetic analysis of AZ82 effects on KIFC1 nucleotide binding and release, little is known about the site in KIFC1 to which AZ82 binds. Crystals of KIFC1 bound to AZ82 have not yet been reported, presumably because stable complex formation requires the motor bound to microtubules, which is technically difficult to obtain.

In lieu of structural data for KIFC1 interactions with AZ82, binding by AZ82 was modeled by positioning the compound in a pocket near the motor ATP-binding cleft that has been proposed to bind a structurally similar inhibitor, GSK923295, to kinesin-7 CENP-E^25^. The proposed binding site also binds several other inhibitors, including monastrol ^26,27^ and ispinesib^28^, to kinesin-5. Other than the overall structural similarity of the KIFC1 inhibitor, AZ82, to the CENP-E inhibitor, GSK923295, evidence for AZ82 binding to the proposed site has not been reported.

Here we report a new crystal structure of human kinesin-14 KIFC1, together with new crystal structures of *Drosophila* Ncd, a KIFC1 orthologue, and human KIFC3, a divergent KIFC1 paralogue, and an analysis of the structural features of the proposed KIFC1 AZ82 inhibitor-binding site. Previous biochemical assays provided evidence that AZ82 is specific for KIFC1 and does not inhibit KIFC3 or other kinesin proteins^24^. The structural differences we observe between KIFC1 and its homologues, KIFC3 and Ncd, and between the two heads of Ncd, which are thought to represent different states of the motor, allow us to reach conclusions regarding the likely binding site of AZ82 in KIFC1.

## Results and Discussion

### KIFC1 and Ncd are kinesin-related homologues

**Figure 1:**
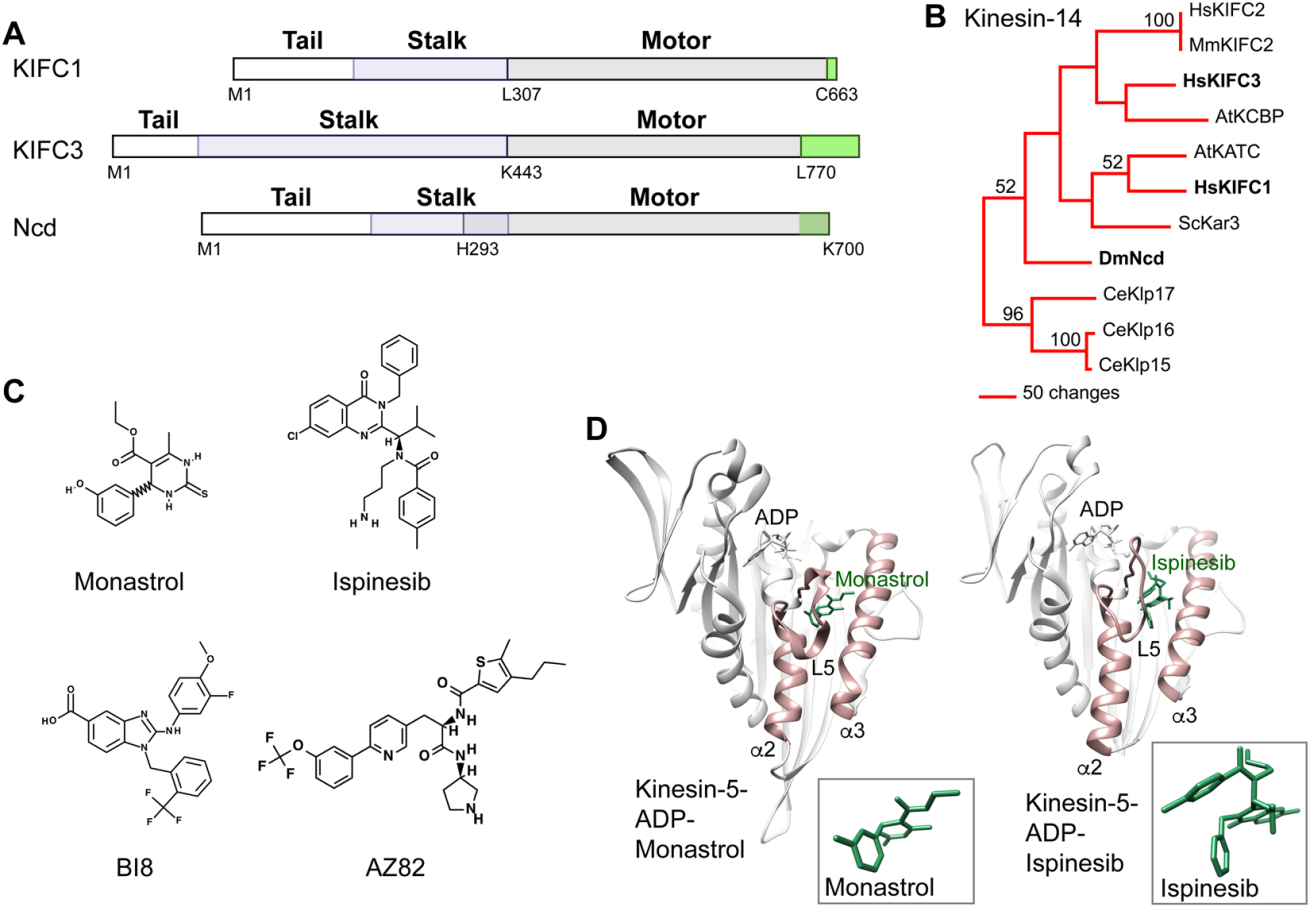
Kinesin proteins and inhibitors. (**A**) Kinesin-14 KIFC1, KIFC3 and Ncd proteins that were crystallized (gray). The KIFC1 and KIFC3 proteins correspond to the conserved motor domain; the Ncd protein includes part of the α-helical coiled-coil stalk (blue) and the C-terminus after the end of the conserved motor domain (green). (**B**) Kinesin-14 family tree showing the three proteins analyzed. (**C**) Kinesin-5 (Monastrol, Ispinesib, BI8) and KIFC1 (AZ82) inhibitors. (**D**) Kinesin-5-ADP-inhibitor crystal structures (Left, PDB 1X88; Right, PDB 4AP0). Insets, conformation of bound inhibitor.

The KIFC1, KIFC3 and Ncd proteins that we analyzed are shown in Fig. 1A, together with a kinesin-14 family tree (Fig. 1B). KIFC1, KIFC2 and KIFC3 group with different proteins in the kinesin-14 tree, rather than forming a single group like the closely related *C. elegans* kinesin-14 Klp15, Klp16 and Klp17 (Fig. 1B). Measurement of branch lengths in the tree indicates that KIFC1 is more closely related to Ncd (326 changes) than KIFC3 (346 changes).

KIFC1 was compared with Ncd and KIFC3 by aligning the protein sequences in the neck, the 14-residue region at the end of the α-helical coiled-coil stalk^29^, and the conserved motor domain. Ncd showed 42.6% sequence identity across 376 residues of KIFC1, corresponding to 345 residues of Ncd. By comparison, KIFC3 showed 45.8% sequence identity across 295 residues of KIFC1, corresponding to 282 residues of KIFC3. Because of the shorter length of homology, the protein sequence similarity score^30^ is lower for KIFC3 than Ncd. By mapping structural elements from an Ncd crystal structure^17^ onto the KIFC1, Ncd and KIFC3 protein sequences, the differences between the motor domains were predicted to be in loops that were longer in KIFC1 than Ncd (L1, L2, L5, L8, L11) or KIFC3 (L5, L8, L11). These findings led us to crystallize the three kinesin-14 proteins to test the hypothesis that KIFC1 differs from Ncd and KIFC3 primarily due to longer loops between conserved structural elements.

### Three new kinesin-14 crystal structures

The new KIFC1 (2.25 Å) and KIFC3 (1.85 Å) structures (Supplementary Fig. S1, Table 1) were determined by the Structural Genomics Consortium. Both proteins were crystallized bound to Mg-ADP and are reported here for the first time. Despite its importance as a potential drug target, this is the first KIFC1 structure to be determined and the only one available to date. KIFC1 and KIFC3 are similar to one another in structure, but show differences in the lengths of loops, as hypothesized above. The largest difference is observed for L11, which is much shorter in KIFC3 (14 residues) than KIFC1 (30 residues), while the adjacent helix α4 is three turns longer in KIFC3 than KIFC1. Despite their relatedness, the kinesin-14 KIFC1 and KIFC3 proteins show different effects with the AZ82 inhibitor: AZ82 binds to KIFC1-MTs and inhibits both the microtubule-enhanced binding of ATP and release of nucleotide by KIFC1, but has no detectable inhibitory effects on KIFC3^24^.

**Table 1:** Diffraction data collection and model refinement.

Kinesin-14 protein	KIFC1	KIFC3	Ncd
**Data collection and reduction**			
Radiation source	APS Beamline 23ID-B	CHESS Beamline F1A	NSRRC Beamline BL13B1
Radiation wavelength [Å]	0.9792	0.9176	1.0
Space group	P 4_1_ 2 2	H 3 2	C2
Cell dimensions a, b, c [Å]	63.7, 63.7, 242.4	187.6, 187.6, 151.4	163.3, 67.2, 94.4
Alpha, beta, gamma [°]	90, 90, 90	90, 90, 120	90, 97.9, 90
Resolution limits [Å]	48.5–2.25 (2.32–2.25)	47.7–1.85 (1.88–1.85)	46.8–2.8 (2.94–2.8)
Unique HKLs	24875 (2203)	86307 (4568)	25309 (3492)
Completeness [%]	99.8 (98.4)	99.7 (100.0)	99.1 (94.7)
R_sym_	0.046 (1.336)	0.048 (0.992)	0.091 (0.533)
I/sigma	29.4 (2.3)	22.2 (2.0)	11.5 (2.7)
CC_1/2_	1.000 (0.945)	1.000 (0.662)	0.998 (0.955)
Redundancy	14.0 (13.8)	6.4 (6.4)	5.8 (5.8)
**Model refinement**			
Refinement resolution [Å]	39.35–2.25	27.08–1.85	46.8–2.8
Reflections used/free	42835/2310^*^	161474/6755^*^	24019/1288
**Number of atoms/average B-factor [Å** ^**2**^ **]**			
Protein (A:B)	2140/84.6	2474/37.6	2831/71.8:2461/94.4
Mg-ADP (A:B)	28/64.6	28/37.2	28/74.4:28/98.7
Water	4/68.4	126/40.7	NA
R_work_/R_free_	0.224/0.265	0.185/0.199	0.235/0.280
RMSD bonds [Å]/angles [°]	0.009/0.9	0.011/0.8	0.008/1.29
Molprobity Ramachandran favored/outliers [%]	97.8/0.0	100.0/0.0	93.0/0.0

^*^Anomalous pairs separated.

The new Ncd dimer structure (2.8 Å) was crystallized in the presence of Mg-AMP·PNP, but was refined with Mg-ADP bound to the nucleotide cleft (Supplementary Fig. S1, Table 1). The dimeric motor shows the stalk and one head, H1, rotated relative to the other head, H2, positioning the two heads asymmetrically with respect to the coiled-coil stalk. The new structure resembles previous mutant Ncd structures that are interpreted to show the motor as it binds to a microtubule in a force-producing conformation^18,31,32^. However, this is the first detailed report of wild-type Ncd in a stalk-rotated conformation.

As in previous stalk-rotated mutant Ncd structures^18,31,32^, the two heads of the new wild-type Ncd structure are thought to represent different states of the motor – H1 shows extensive interactions with the end of the coiled-coil stalk, or neck^33,34^, while H2 shows disrupted neck interactions and appears positioned to bind to a microtubule^18,31,32^. The two heads differ from one another in the conformation of loops L8, L10 and L12, which are ordered in H1, but disordered in H2. In addition, several helices differ in length in H1 compared to H2. These include helix α3, which is longer by one turn in H2, and helices α4 and α6, which are shorter at both ends in H2 by half a turn or a full turn, respectively. There is also a conformational change in loop L13 between the two heads that confirms interpretations from a mutant structure, NcdG347D, regarding changes that occur in L13 and their effects in inducing stalk rotation^32^.

Comparison of KIFC1 to Ncd shows that the two motors are structurally very similar to one another with differences in loops, which are longer in KIFC1 than Ncd H1 or H2, as predicted above. In addition, helices in Ncd H1 (α1, α3, α4, α6) and H2 (α3, α4, α5, α6) differ in length from KIFC1. The structural relatedness between KIFC1 and the other two kinesin-14 motors led us to compare the known inhibitor binding sites in the new structures to gain insights into the structural features that allow AZ82 to inhibit KIFC1, but not KIFC3 or other kinesins^24^.

### Kinesin L5/α2/α3 inhibitor binding pocket

Loop L5, one of the loops that is longer in KIFC1 than in Ncd or KIFC3, has been shown previously to be important for binding by specific inhibitors to kinesin-5. The kinesin-5 inhibitors differ in size and structure, and include monastrol and ispinesib (Fig. 1C). However, crystal structures show that they bind to the same site in kinesin-5, near the nucleotide-binding cleft and adjacent to L5 (Fig. 1D)^26,28^. Moreover, mutant analysis implicates binding by the kinesin-7 CENP-E inhibitor, GSK923295, to the CENP-E L5 site^25^, despite the large size of GSK923295 (Supplementary Fig. S2) compared to monastrol and ispinesib (Fig. 1C).

The kinesin inhibitor-binding site near the nucleotide-binding cleft consists of a pocket formed by helices α2 and α3, which is enclosed by L5 (Fig. 1D). Monastrol fits in the kinesin-5 pocket with the hydroxyphenyl ring rotated perpendicular to the pyrimidine ring (Fig. 1D; space-filled monastrol dimensions, ~17 × 16.2 Å). The compound is held in place on either side by residues of helix α2 (P137) and α3 (L214, A218), and enclosed by residues of loop L5 (G117, E118, R119, W127). In the case of ispinesib, the methyl-benzyl and benzyl rings have rotated into the plane above or perpendicular to the quinazolinone, respectively, folding into a compact, stacked conformation (Fig. 1D; space-filled ispinesib dimensions, ~24.8 × 19.2 Å). This allows the molecule, which is bulkier and approximately twice the molecular mass of monastrol, to fit into the same cleft as monastrol. Ispinesib is bound by the same residues of α2, α3 and L5 as those that bind monastrol.

**Figure 2:**
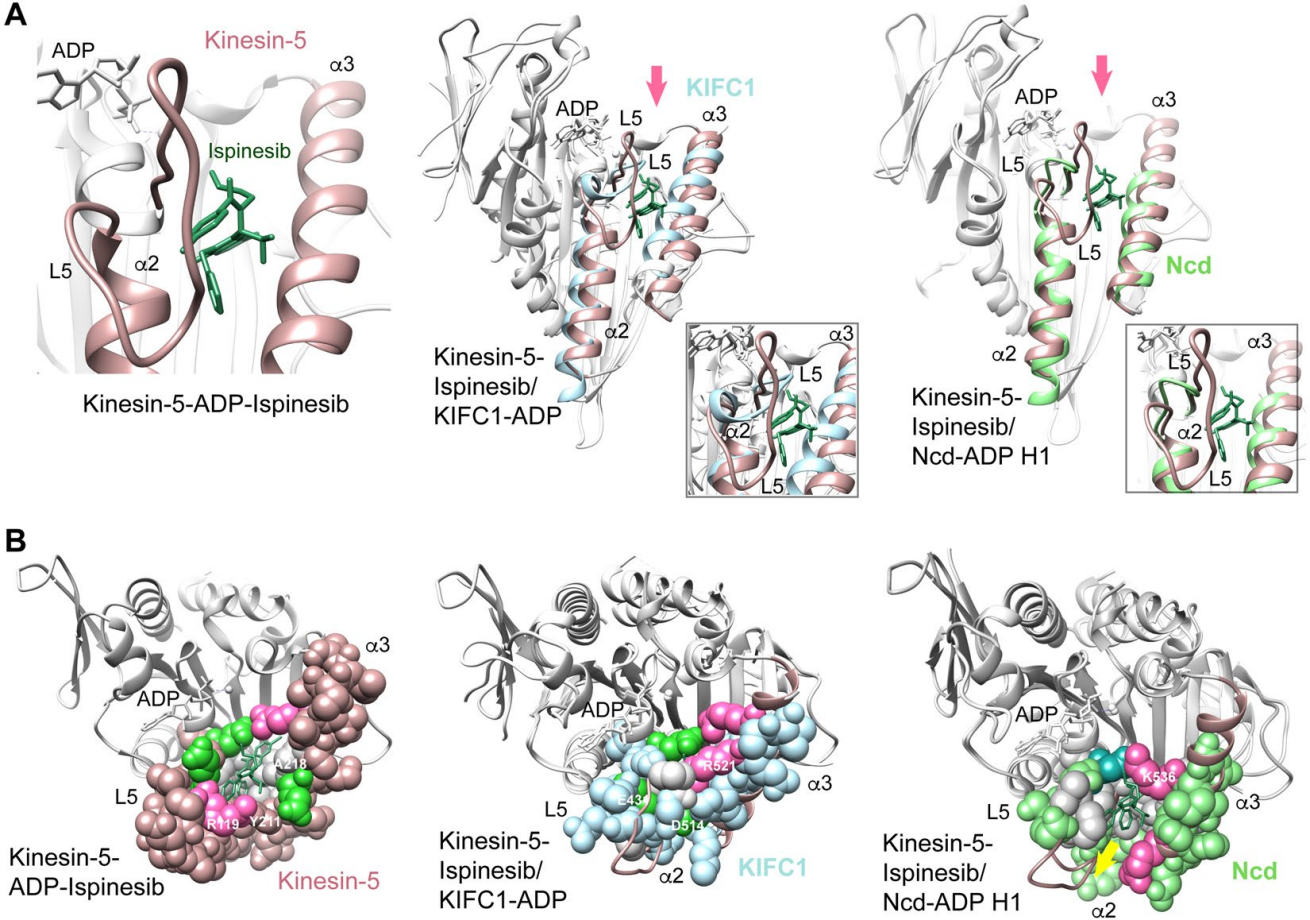
Kinesin L5/α2/α3 inhibitor binding site. (**A**) Ribbon diagrams. Left, kinesin-5-ADP-ispinesib (PDB 4AP0; L5/α2/α3, dark pink; ispinesib, dark green). Center, KIFC1-ADP (PDB 5WDH; L5/α2/α3, pale blue) superposed with kinesin-5-ADP-ispinesib. Right, Ncd-ADP H1 (PDB 5W3D; L5/α2/α3, green) superposed with kinesin-5-ADP-ispinesib. Insets, close-up view of binding site. Arrows, line of view for structures in B. (**B**) Space-filled L5/α2/α3 pocket, viewed from the top, as indicated by arrows in A. Hydrophobic residues, gray; basic residues, hot pink; acidic residues, green or dark aqua (Ncd). ADP, white. Arrow (yellow), opening at bottom of pocket.

The L5/α2/α3 pocket was the first inhibitor binding site reported for the kinesins and has been reported to bind a wide range of compounds with differing primary groups^35^. Binding by inhibitors induces structural changes^26,28^ causing kinesin-5 to assume an inhibitor-bound conformation^36^ distinct from the AMP·PNP-bound conformation^37,38^. Superposition of kinesin-5-ADP structures with and without bound ispinesib shows remodeling of loop L5 upon ispinesib binding and an increased tilt of helix α3 away from helix α2, opening the pocket (Supplementary Fig. S2). Similar changes are observed upon monastrol binding^26,38^. However, the L5/α2/α3 pocket, lined at the bottom and one side with hydrophobic residues (Supplementary Fig. S2), is present in kinesin-5-ADP before ispinesib binding, indicating that the compound binds to and induces changes in a pre-existing pocket, rather than creating a new pocket in the motor. The changes in the kinesin-5 L5/α2/α3 pocket after inhibitor binding (Fig. 2A Left) include an increase in hydrophobic residues (space-filled, gray) at the bottom of the pocket, and moving together of the charged residues at the top (Fig. 2B Left; acidic residues, green; basic residues, hot pink).

Inhibitors that bind to the L5/α2/α3 pocket cause allosteric, ATP noncompetitive effects – binding by the inhibitors affects ADP release by the motor, but the inhibitors do not compete with ATP for binding or prevent ATP binding to the motor^27,39,40^. Because of the structural evidence implicating the L5/α2/α3 pocket as a binding site for kinesin-5 inhibitors, as well as evidence implicating binding to the pocket by a kinesin-7 inhibitor, the AZ82 inhibitor of kinesin-14 KIFC1 (Fig. 1C) was proposed to bind at the site^24^.

In order to evaluate the ability of the KIFC1 L5/α2/α3 pocket to bind small molecules, we superposed our KIFC1-ADP crystal structure with kinesin-5-ADP-ispinesib. Viewed from the nucleotide-binding side of the motor, the ribbon diagram of the superposed structures shows the KIFC1 helix α3 tilted inward towards helix α2, reducing the size of the pocket in KIFC1 (Fig. 2A Center; KIFC1 L5/α2/α3, pale blue). In addition, the shorter KIFC1 loop L5 (12 residues) compared to kinesin-5 (19 residues) is positioned over the bound ispinesib, covering the compound (Fig. 2A Center, inset). When rendered as space-filling residues, the KIFC1 L5/α2/α3 pocket is enclosed and not visible when viewed from the top (Fig. 2B Center), although there is a small opening at the lower right side (not visible in Fig. 2B). The pocket itself is greatly reduced in size and only large enough to fit the methyl-phenyl ring of ispinesib, but not the phenyl ring and quinazolinone group. There are clashes of ispinesib with several residues of KIFC1 helix α3 due to the differences in the angle and residues that make up the KIFC1 α3 helix compared to kinesin-5.

A critical residue of KIFC1 relevant to formation of the L5/α2/α3 pocket is R521 of helix α3, which is positioned under loop L5 (Fig. 2B, Center, hot pink). Interaction of the side chain of R521 with other residues stabilizes L5 and maintains the closed conformation of the potential binding pocket, disfavoring binding by small molecule at the site. The corresponding residue, A218, in kinesin-5 (Fig. 2B, Left, gray) does not form these L5-stabilizing interactions because of its short side chain. In kinesin-5, the conformation of L5 is maintained by interactions between loop L5 R119 and helix α3 Y211 (Fig. 2B, Left), which undergo rearrangement upon small molecule binding.

By comparison to KIFC1, the L5/α2/α3 pocket is visible from both the side and top in the KIFC1 homologue, Ncd (Fig. 2A,B Right; Ncd H1 L5/α2/α3, green). The pocket is lined at the bottom and one side with hydrophobic residues (space-filled, gray) and enclosed by charged residues (acidic, aqua; basic, hot pink) at the back. However, loop L5 of Ncd is shorter (8 residues) than in kinesin-5 or KIFC1, and leaves an opening at the lower front of the pocket, as well as the top, through which the compound could escape (Fig. 2B Right, arrow). In addition, residues of Ncd helix α3 would interfere with the compound entering as deeply into the back of the pocket as in kinesin-5, again disfavoring stable binding. Ncd K536 (Fig. 2B Right, hot pink), the residue corresponding to KIFC1 R521, which maintains the closed conformation of the potential binding pocket, is positioned away from loop L5. The other Ncd head, H2, which is thought to assume a conformation resembling the motor bound to a microtubule, shows an L5/α2/α3 pocket similar to that of Ncd H1, although R529 (Fig. 2B Right, near arrow) has moved up, partially closing the opening – the charged arginine residue could potentially contribute to stable binding by a small molecule, depending on its interactions.

In the case of the KIFC1 human homologue, KIFC3, an L5/α2/α3 pocket is visible (Supplementary Fig. S3), that again is smaller than in kinesin-5, similar to the size of the pocket in Ncd. There is an opening near the bottom front of the pocket (Supplementary Fig. S3), as well as the top. In KIFC3, the side chain of H630, corresponding to KIFC1 R521, is not long enough to interact with L5. There are no other interactions between loop L5 and helix α3, consistent with the open conformation of the potential binding site. This will also disfavor the binding of small molecules.

Thus, the motor-ADP conformation of all three proteins, KIFC1, Ncd and KIFC3, would disfavor binding to the L5/α2/α3 pocket by a small molecule of the same shape and size as ispinesib, although conformational changes in the Ncd pocket during the ATP hydrolysis cycle could potentially lead to small molecule binding. In particular, the pocket in KIFC1 is greatly reduced in size compared to kinesin-5-ADP-ispinesib or kinesin-5-ADP and closed at the top by loop L5 and helix α3 residues, and not readily accessible. KIFC1 loop L5 is shorter in length than in kinesin-5 and has a B factor (70.0 Å^2^) lower than the overall protein (Table 1, 84.6 Å^2^), indicating that it is less mobile than other regions of the protein. In addition, L5 in other kinesins does not undergo large structural changes in different states (Supplementary Fig. S2) – thus, restructuring of L5 is unlikely to open the KIFC1 L5/α2/α3 pocket in other nucleotide states. The small opening to the pocket at the lower front and the negatively charged residues (E431, D514) positioned there (Fig. 2B, Center) would inhibit binding by hydrophobic compounds.

By contrast, the binding cleft can be observed in Ncd, although it is not as deep as in kinesin-5. There is also an opening at the lower front that is not covered by loop L5, although partial closure of the opening by movement of an arginine could lead to stable binding by inhibitors. Finally, the KIFC3 L5/α2/α3 binding pocket is smaller than in kinesin-5, similar in size to Ncd, and does not appear large enough to accommodate a molecule the size and shape as ispinesib without opening of the cleft upon inhibitor binding. The KIFC3 pocket is also open at the lower front, as in Ncd, and it is uncertain whether charged or other residues could undergo structural changes to close the opening and prevent escape of inhibitors from the pocket.

### Kinesin α4/α6 inhibitor binding site

**Figure 3:**
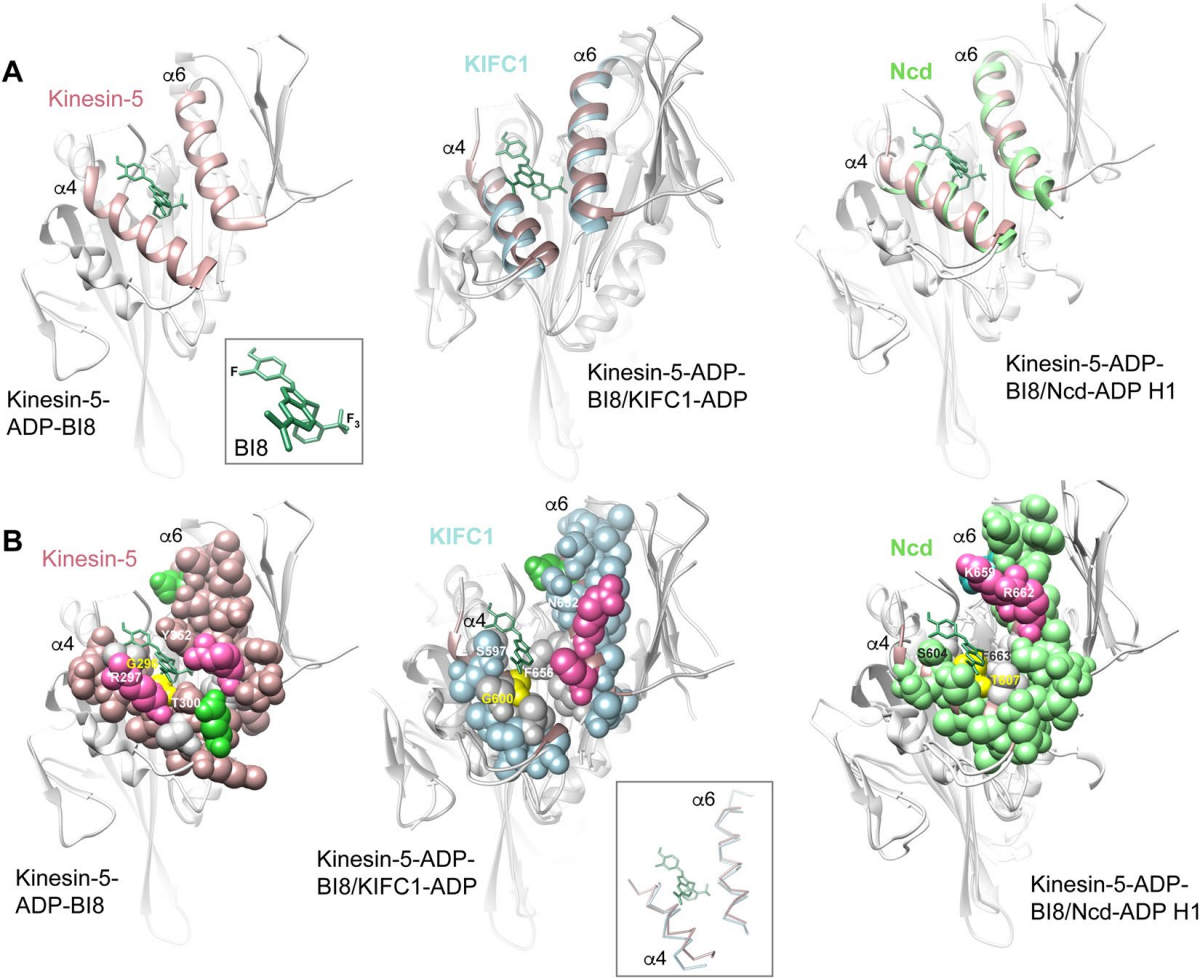
Kinesin α4/α6 inhibitor binding site. (**A**) Ribbon diagrams. Left, kinesin-5-ADP-BI8 (PDB 3ZCW; α4/α6, dark pink; BI8, dark green). Inset, bound BI8 conformation. Center, KIFC1-ADP (PDB 5WDH; α4/α6, pale blue) superposed with kinesin-5-ADP-BI8. Right, Ncd-ADP H1 (PDB 5W3D; α4/α6, green) superposed with kinesin-5-ADP-BI8. (**B**) Space-filled α4/α6 cleft. Hydrophobic residues, gray; basic residues, hot pink; acidic residues, green or dark aqua (Ncd). Kinesin-5 G296 and corresponding residues, yellow (KIFC1, G600; Ncd, T607). Center inset, carbon backbone trace of the α4/α6 cleft protein chains.

A second kinesin inhibitor-binding site on the opposite side of the motor as the L5/α2/α3 pocket has been reported recently. This site at the motor microtubule-binding surface was first detected for a new class of kinesin-5 biaryl inhibitors that are ATP competitive, but do not bind to the motor nucleotide-binding cleft. Mutational analysis and photo-affinity labeling established the site of binding by these inhibitors to a cleft formed at the interface of helix α4 with helix α6^41^. Structural evidence that inhibitors bind with high affinity to the newly identified site was obtained by crystallization of a benzimidazole derivative, BI8 (Fig. 1C), bound to kinesin-5^42^. The kinesin-5-ADP-BI8 crystal structure (Fig. 3A,B Left) contains two molecules of BI8, one bound at the L5/α2/α3 site and the other bound at the new α4/α6 site (space-filled BI8 dimensions, ~21.8 × 15.7 Å). Isothermal titration calorimetry competition assays and surface plasmon resonance analysis showed that the BI8 at the L5/α2/α3 site was weakly bound, but the BI8 at the second site was tightly bound. A more recent report shows binding to kinesin-5 by a new biphenyl compound, smaller than BI8, only at the α4/α6 site^43^.

Differences in conformation between kinesin-5-ADP and kinesin-5-ADP-BI8 are again observed for binding at the α4/α6 site, but the binding cleft is visible in the motor-ADP (Supplementary Fig. S4). Helix α6 of kinesin-5-ADP-BI8 has moved away from helix α4, changing its tilt and widening the cleft. Three residues that are thought to be important for BI8 binding to the kinesin-5 α4/α6 site are Y352, R297 and T300 (Fig. 3B, Left)^42^. Both Y352 (not visible in the view shown in Supplementary Fig. S4) and T300 are buried deeper in the kinesin-5-ADP cleft, which has widened at the bottom to permit binding by the BI8 inhibitor in kinesin-5-ADP-BI8.

Superposition of kinesin-5-ADP-BI8 with KIFC1-ADP (Fig. 3A,B Center; KIFC1 α4/α6, pale blue) shows that the KIFC1 α4/α6 pocket is wider (Fig. 3B, inset) and not as deep as in kinesin-5, forming an open cleft. The residues that interact with BI8 differ in KIFC1 from kinesin-5, and the compound is positioned to one side of the pocket, although sufficient space in the cleft appears to exist for binding by a small molecule of similar shape and size as BI8. The cleft is lined with hydrophobic residues on one side and the bottom (Fig. 3B Center; space-filled, gray) and charged residues (acidic, green; basic, hot pink) on the other side. Binding to the cleft by a small molecule could potentially be stabilized by hydrophobic and/or charge:charge interactions, or by movement of helix α6 that closes the cleft. Binding to KIFC1 could occur first^24^ and subsequent binding of the complex to microtubules could prevent the molecule from diffusing out of the cleft – a crystal structure of an inhibitor bound to the motor α4/α6 cleft^43^ without microtubules indicates that this sequence of events may occur in the case of another inhibitor that affects a microtubule-bound kinesin motor.

Kinesin-5-ADP-BI8 superposition with Ncd-ADP (Fig. 3A,B Right; Ncd H1 α4/α6, green) shows a similarly widened α4/α6 pocket as in KIFC1 with one side and the bottom of the cleft lined by hydrophobic residues. The residues lining the other side of the cleft differ from KIFC1 in that they include a lysine, K659, with the adjacent arginine, R662. These positively charged residues also differ from the kinesin-5 residues involved in binding to BI8. The charges due to the lysine and arginine may mediate stable binding by small molecules to the pocket. Ncd H2 shows movement of helices α4 and α6 towards each other, narrowing the opening to the cleft. In particular, Ncd H2 K659 has moved towards S604, partially enclosing the opening of the cleft, while R662 has moved outward, tilting away from F663. These movements could facilitate stable binding by a small molecule to the Ncd α4/α6 pocket.

Kinesin-5-ADP-BI8 superposed with KIFC3-ADP shows that KIFC3 helix α4 is longer than kinesin-5 by three turns (Supplementary Fig. S4), elongating the α4/α6 pocket. Several residues of the KIFC3 α4/α6 pocket are present in similar positions as in kinesin-5, including a tyrosine, Y757, that in kinesin-5 (Y352) is thought to stack face-to-face with the benzimidazole ring of BI8.

**Figure 4:**
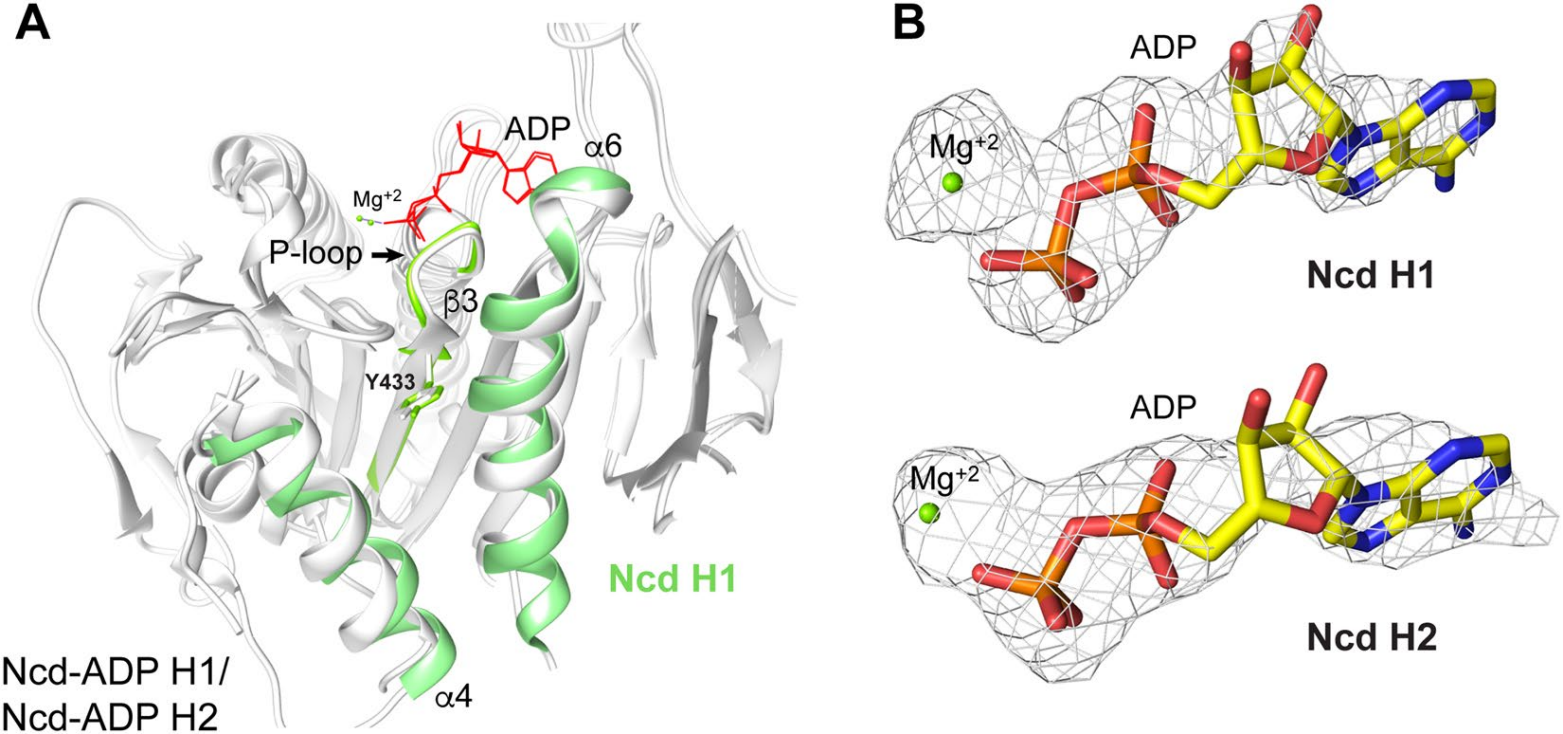
Ncd Y433 and nucleotide release. (**A**) Ncd α4/α6 binding cleft. Ncd-ADP H1 (PDB 5W3D; α4/α6, green) superposed with H2 (white). Ncd H1 β3, Y433 and P-loop, yellow green; H1 and H2 Mg^+2^, yellow green; ADP, red. (**B**) F_o_–F_c_ difference maps of ADP bound to Ncd H1 and H2. Density for the ADP ribose moiety in H2 is less well defined than in H1 and the magnesium ion has moved further away from the beta phosphate in H2 than in H1.

Kinesin-5 Y104 at the end of strand β3 (not visible in the view shown in Fig. 3B, but see Fig. 4A for Ncd Y433) makes an important contribution to the α4/α6 binding site by forming stacking interactions with the trifluoromethyl phenyl group of BI8^42^ or the 3-fluoro-4-trifluoromethyl phenyl group of PVZB1194, another biaryl inhibitor that binds to the α4/α6 cleft^43^. The residue corresponding to kinesin-5 Y104 is invariant in the kinesin motors – it is present in the motif, IFA**Y**GQT, adjacent to the nucleotide binding cleft, or P loop (Fig. 4A). Interactions of Y104 with inhibitors bound to the α4/α6 cleft are interpreted to induce conformational changes that destabilize the P loop, disrupting stable ATP binding^43^. This would cause the inhibitors to show competition with ATP for motor binding, explaining their ATP competitive effects.

Strikingly, Ncd H2 strand β3 has moved upward from its buried position in H1 – it does this by gaining a residue at its C terminus (G434), as well as its N terminus (N427). Ncd Y433, which corresponds to kinesin-5 Y104, remains positioned near the bottom of the cleft (Fig. 4A). The transition of G434 to a β-strand in Ncd head H2 β3 shortens the adjacent P-loop by a glycine residue, which may make the P-loop less flexible and more likely to release bound nucleotide. This is consistent with the reduced density of ADP bound to head H2 compared to head H1 (Fig. 4B), supporting the interpretation that head H2 is releasing ADP, representing a microtubule-binding conformation^18,31,32^. Movement upward of β3 is not observed in kinesin-5-ADP-BI8 compared to kinesin-5-ADP, and could be a characteristic of the kinesin-14 motors. If so, a similar change in KIFC1 β3 without affecting the position of the invariant tyrosine (KIFC1 Y409) could stabilize binding by inhibitors that involve interactions between the tyrosine and inhibitor, but still result in ATP release, causing ATP competitive effects.

Thus, the KIFC1, Ncd and KIFC3 α4/α6 clefts differ in shape from kinesin-5, appearing wider and more open in all three kinesin-14 proteins. Binding by an inhibitor of the same size and shape as BI8 could occur, but the inhibitor would probably diffuse out of the cleft unless it is prevented from doing so by hydrophobic or charge:charge interactions, by closure of the cleft upon inhibitor binding, or by subsequent microtubule binding. Comparison of the two heads of the new wild-type Ncd dimeric structure indicates that changes in the cleft occur in Ncd and may also occur in other kinesin-14 motors. The positions of the hydrophobic and charged residues and differences in the positions of these residues among the three kinesin-14 motors could confer binding specificity for different compounds.

### AZ82 inhibitor of KIFC1

The KIFC1 inhibitor, AZ82, was discovered in a high throughput screen of compounds that inhibit KIFC1 microtubule-stimulated ATPase activity^24^. AZ82 is a biaryl compound that contains a trifluoromethoxy-benzyl group similar to the trifluoromethyl-benzyl group of BI8 (Fig. 1D). Assays of AZ82 showed that the compound specifically inhibits KIFC1 microtubule-stimulated ATP hydrolysis and that inhibition of KIFC1 by AZ82 is ATP competitive and microtubule noncompetitive. Assays with other kinesins, including the KIFC1 human homologue, KIFC3, showed that AZ82 is highly specific for KIFC1 and has no detectable activity against other kinesins that were tested^24^.

Analysis of the KIFC1 and Ncd crystal structures shows differences in both of the known kinesin inhibitor binding sites, as well as differences compared with KIFC3. In particular, the KIFC1 L5/α2/α3 site does not appear to be well suited for inhibitor binding because the tilt of helix α3 and the residues that comprise it almost fill the pocket that is formed, making it too small for a molecule the size of AZ82. The pocket could open to accommodate binding by AZ82, but the structural changes observed in kinesin-5 upon binding by ispinesib, which is smaller than AZ82, would probably not open the KIFC1 pocket sufficiently for binding by AZ82. The L5/α2/α3 pocket is present in Ncd and KIFC3, but the openings at the side and top are likely to facilitate diffusion of the compound out of the site unless closure of the L5/α2/α3 pocket occurred, which appears possible in Ncd by comparison of head H2 with H1. By contrast, the α4/α6 cleft is present in all three kinesin-14 motors and could bind AZ82, although it is wider and more open than in kinesin-5. However, closure of the cleft and remodeling of the pocket could occur upon compound binding, based again on the structural changes observed in the Ncd head H2 α4/α6 cleft, compared to H1.

A critical residue difference in the α4/α6 binding pocket among the kinesin-14 proteins is the one corresponding to kinesin-5 helix α4 G296 – this residue (shown in yellow in Fig. 3B and Supplementary Fig. S4) is conserved in KIFC1 (G600) and KIFC3 (G705), but changed to T607 in Ncd. The T607 side chain would make the binding of AZ82 unfavorable at the Ncd α4/α6 pocket, if the binding involves the same residue interactions as BI8 binding to kinesin-5. The conservation of kinesin-5 G296 in both KIFC1 and KIFC3, but the specificity of AZ82 for KIFC1, argues that binding of AZ82 to KIFC1, if it occurs at the α4/α6 site, differs from that of BI8 to kinesin-5.

Three features of AZ82 indicate that its binding is more likely to the KIFC1 α4/α6 cleft than the L5/α2/α3 pocket, as proposed previously^24^. The first and probably most significant is that the effect of AZ82 on KIFC1 has been reported to be ATP competitive^24^. Competition by the AZ82 inhibitor with ATP for KIFC1 binding disfavors binding to the L5/α2/α3 pocket, which has not been reported to affect ATP binding by kinesin-5, despite the varied compounds that have been found to bind to this site^35^. Instead, the inhibitors that bind to the L5/α2/α3 pocket cause ATP noncompetitive effects^27,39,40^. Alternatively, AZ82 could produce ATP competitive effects by binding to the nucleotide-binding cleft, although binding by specific kinesin inhibitors to the P-loop has not yet been observed. Moreover, the shape of the AZ82 molecule argues against this possibility, although it does not exclude it, as the molecule could adopt a conformation that fits into the cleft. If AZ82 does not bind to the nucleotide-binding cleft, the α4/α6 pocket is a good candidate for its binding site, given that binding to this site by another kinesin inhibitor, PVZB1194, has been reported to cause allosteric changes in the motor that affect ATP binding^43^. Further, binding interactions of PVZB1194 reveal a mechanism by which its binding causes ATP competitive effects in kinesin-5 – its interactions with an invariant tyrosine adjacent to the highly conserved P loop destabilize nucleotide binding to the motor. This effect is dependent on the proximity of the binding site to the P loop. Because of this, it seems unlikely that other binding sites in the kinesin motors exist with this property.

The second and third features that may contribute to AZ82 binding to the α4/α6 pocket is that it contains both biaryl and trifluoro groups, resembling other molecules that have been found to bind to the site. Ring-stacking interactions between the compounds and the invariant tyrosine adjacent to the P-loop (Y104 in kinesin-5) disrupt ATP binding. Although the AZ82 biaryl and trifluoro groups may not determine the ability of the compound to bind to the α4/α6 cleft, they indicate that the molecular conformation of AZ82 resembles that of similar compounds, so that it is potentially capable of binding to the site. Note that other molecules that bind to the site may not show ATP competitive effects unless they interact with the invariant tyrosine. Those that do so, however, are expected to be ATP competitive.

**Figure 5:**
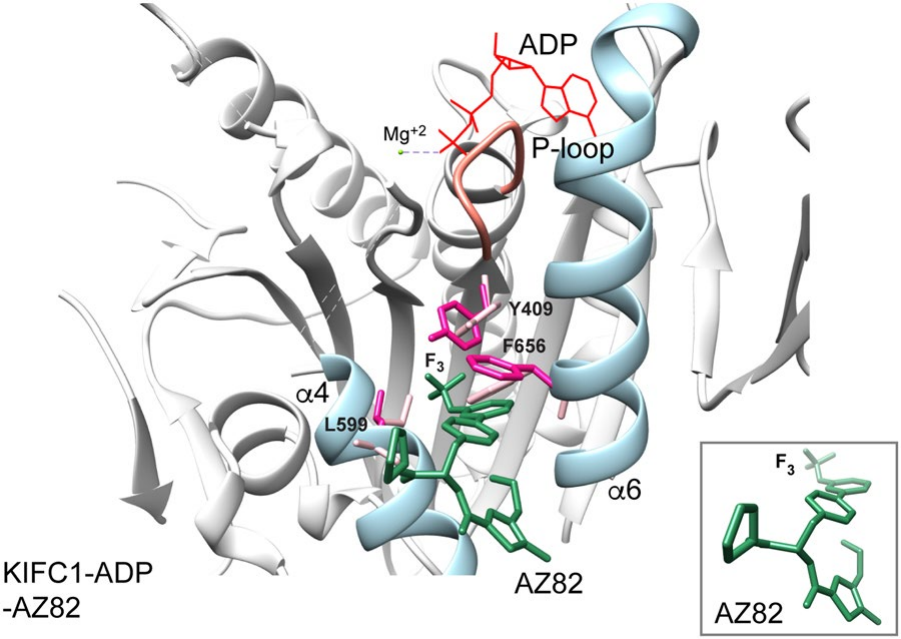
AZ82 docked into the KIFC1 (PDB 5WDH) α4/α6 cleft. AZ82 binding to the KIFC1 α4/α6 cleft gave a predicted free energy change of ΔG = −8.1 kcal/mol. The predicted free energy changes for AZ82 binding to KIFC3 (ΔG = −5.4 kcal/mol) and Ncd (H1, ΔG = −7.9 kcal/mol; H2, ΔG = −6.5 kcal/mol) indicated less favorable binding. The lower predicted free energy change for AZ82 binding to Ncd head H2 than H1 indicates that the drug is unlikely to specifically inhibit Ncd motor-MT activity, as observed for KIFC1. In addition, structural features of AZ82 binding to KIFC3 and Ncd (see Supplementary Fig. S5 for Ncd H1), indicate that the drug may not be stably bound. KIFC1 residues Y409, L599 and F656 are shown as stick models before (pale pink) and after (dark pink) docking. AZ82, dark green; KIFC1 helices α4 and α6, pale blue; P-loop, coral; ADP, red; Mg^+2^, yellow green. Inset, AZ82 conformation after docking.

In order to further evaluate the KIFC1 α4/α6 cleft as a potential binding site for the AZ82 inhibitor, we performed docking calculations of AZ82 onto KIFC1. AZ82 (PubChem CID 91885438) was energy minimized and free energy changes were calculated after complex formation with KIFC1 using PyRx^44^. The KIFC1 conformation with the highest predicted AZ82 free energy change shows movement of residues, including Y409, L599 and F656, to form the binding site (Fig. 5). The AZ82 trifluoromethoxy group contacts the Y409 side chain and the AZ82 pyridine moiety is stacked against the F656 side chain. The AZ82 trifluoro (F_3_) group is buried in the pocket, as in kinesin-5-BI8^42^ and kinesin-5- PVZB1194^43^. Movement of Y409 could destabilize the adjacent P-loop, causing release of nucleotide upon AZ82 binding. The docking shows a potential conformation of the KIFC1-AZ82 complex that could explain the ATP-competitive effects caused by AZ82 binding to KIFC1.

Docking controls of AZ82 into KIFC3 and Ncd showed predicted free energy changes that were lower than for KIFC1 (Fig. 5), although the predicted free energy change for Ncd head H1 (ΔG = −7.9 kcal/mol) was close to that for KIFC1 (ΔG = −8.1 kcal/mol). However, the predicted free energy change for Ncd head H2 (ΔG = −6.5 kcal/mol), the head positioned to bind to the microtubule, was lower than for H1, in contradiction to the observation that the drug acts on the microtubule-bound motor – this indicates that AZ82 binding is unlikely to specifically inhibit Ncd-MT activity, as observed for KIFC1.

In addition, AZ82 was bound to a shallow groove in KIFC3, as well as in Ncd H1 and H2, because binding by the drug did not cause the side chain of a conserved phenylanine (F761 in KIFC3, F663 in Ncd; see Supplementary Fig. S5 for Ncd H1) that lines the α4/α6 pocket to flip up, opening the pocket, as it does for F656 in KIFC1 (Fig. 5). The movement of F656 allows the hydrophobic AZ82 F_3_ group to become buried deep in the KIFC1 α4/α6 pocket, which is likely to stabilize AZ82 binding. By contrast, the shallower binding to KIFC3 and Ncd may cause the drug to be released when the motor interacts with a microtubule.

### Other kinesin inhibitor binding sites

Other possible binding sites for inhibitors in the kinesin motor domain have been proposed, based on molecular dynamics simulations^45^. One of these involves a pocket between helix α5 and the central β-sheet near the neck linker, a structural element present in plus-end kinesins consisting of two β-strands that connect the coiled-coil stalk to the conserved motor domain. The neck linker is thought to produce force when it docks onto the motor domain^8^. It is not present in the kinesin-14 motors because of differences in their overall structure. Instead, the kinesin-14 motor domain is directly connected to the stalk, resulting in a different force-producing mechanism compared to plus-end kinesins^38^. Loop L13 is thought to mediate stalk rotation in kinesin-14 Ncd: L13 S639 hydrogen bonds to G347D at the end of the stalk in the Ncd G347D mutant^32^, disrupting neck-motor interactions, including N340-K640 – these interactions maintain stalk position relative to the motor domain and when disrupted, the stalk rotates^10,32^. The new wild-type Ncd crystal structure shows movement of G347 towards L13 S639, as observed in Ncd G347D (see Fig. 4 of ref.^32^), and disruption of N340-K640 in head H2, indicating that this interaction is also critical in wild-type Ncd. A pocket in head H1 near the base of L13, formed by the C-terminus of helix α4 and junction of the neck with the Ncd motor domain, may be analogous to the binding site near the neck linker proposed for plus-end motors^45^. If so, binding by compounds to the site would probably not be ATP competitive, given its distance from and apparent lack of interactions with the nucleotide-binding cleft of the motor.

### Perspectives

Considerable interest has focused on the kinesin microtubule motor proteins as potential targets for therapeutic agents against cancers because of their essential roles in mitosis, although this has also limited their effectiveness due to side effects in normally dividing cells. KIFC1, a human kinesin-14 motor, is an apparent exception, in that it is nonessential in almost all normal human cells, but many cancer cells are dependent on the motor for viable division. Although new small molecule inhibitors of KIFC1 have been discovered, their development has been delayed by the inability to obtain motor-inhibitor crystal structures, due to the necessity of crystallizing the technically difficult-to-obtain motor-MT-inhibitor complex. Here we report three new kinesin-14 crystal structures and a comparative analysis that leads us to conclusions regarding the probable site of inhibitor binding in the motor.

Structural analysis shows that the first identified kinesin inhibitor-binding site, the L5/α2/α3 pocket, is probably not large enough in KIFC1 to fit a small molecule larger than a methyl-phenyl ring, but that a second site^41–43^, the α4/α6 cleft, is present in all three kinesin-14 motors that were analyzed. Movements of residues between the two heads of kinesin-14 Ncd, which is closely related to KIFC1, show that conformational changes occur that could stabilize binding to the α4/α6 site. The ATP competitive effects demonstrated by inhibitors bound to this site are consistent with the effects of a potent KIFC1 inhibitor, AZ82^24^. The properties of this site predict that other biaryl trifluoro aromatic compounds will be potent specific inhibitors of KIFC1. Compounds that bind to this site in KIFC1 and interact with or cause movements of KIFC1 Y409 of strand β3 are predicted to show ATP competitive effects by destabilizing the adjacent P loop.

Finally, the ability to crystallize kinesin-5 bound to an ATP-competitive inhibitor, PVZB1194, without nucleotide^43^, means that it should be possible to crystallize other kinesin motor-inhibitor complexes in the no-nucleotide state of the cycle. Kinesin-14 Ncd has already been crystallized in a conformation that is thought to resemble a microtubule-bound motor^18,31,32^. If KIFC1 and other kinesins can be crystallized in the same conformation, structural analysis of the ATP-competitive inhibitors of the motors should be possible. If so, this will lead to further insights into kinesin motor function, as well as information about the effects of specific inhibitors and their mechanism of function.

## Methods

### Phylogenetic analysis

Trees were built with the maximum parsimony program, PAUP^46^, using the heuristic search method with random stepwise addition and tree bisection-reconnection branch swapping, as described previously^15,47,48^. The optimized alignment (available on request) contained 11 kinesin-14 proteins and 2 outgroup proteins, CfDSK1 and ScSMY1. A single optimal tree was found. The tree was rooted using ScSMY1, a divergent kinesin protein from budding yeast, as the outgroup taxon. Bootstrap analysis (1,000 replicates) was performed using the heuristic search method with simple stepwise addition and ScSMY1 as the reference taxon. Abbreviations and accession numbers for the proteins that were analyzed are the following: *Arabidopsis thalia* KATC (AtKATC, D21138) and KCBP (AtKCBP, L40358); *Caenorhabditis elegans* Klp15 (CeKlp15, U80450), Klp16 (CeKlp16, Z81048) and Klp17 (CeKlp17, AB031233); *Cylindrotheca fusiformis* DSK1 (CfDSK1, U51680); *Drosophila melanogaster* Ncd (DmNcd, X52814); *Homo sapiens* KIFC1 (HsKIFC1, NM_002263), KIFC2 (HsKIFC2, NM_145754) and KIFC3 (HsKIFC3, BC001211); *Mus musculus* KIFC2 (MmKIFC2, D49545); *Saccharyomyces cerevisiae* Kar3 (ScKar3, M31719) and SMY1 (ScSMY1, M69021).

### Protein purification and crystallization

KIFC1 motor domain (N307-C663 with T368P) was cloned in *pFBOH-LIC* (a gift of Cheryl Arrowsmith, SGC, Toronto, CAN; Addgene #26099) and expressed in Hi-Five insect cells (Gibco). T368P is a PCR-induced mutation in loop L2, which is disordered in KIFC1, as well as in Ncd H1 and H2, and is not expected to greatly affect motor structure. KIFC3 motor domain (K443-L770) was cloned in *p28a-LIC* (a gift of Peter Loppnau, SGC, Toronto, CAN; GenBank EF442785) and expressed in *E. coli BL21-CodonPlus (DE3)-RIL* (Agilent Technologies). KIFC1 was purified by Ni-NTA and Superdex 75 chromatography and KIFC3 by DE52, HisTrap and Superdex chromatography. KIFC1 (16 mg/ml + 5-fold molar excess Mg-ADP) was mixed with 3.5 M NaCl + 0.1 M Bis-Tris propane, pH 7.5 and KIFC3 (35 mg/ml) was mixed with 1.2 M sodium citrate, 0.1 M Hepes, pH 7.5 for crystallization at 18 °C. KIFC1 crystals grew in one day and KIFC3 crystals in two days. Both crystals were harvested in cryoprotection buffer consisting of mother liquor + 20% glycerol and flash-frozen in liquid nitrogen.

Dimeric Ncd (MGSM-H293-K700) was expressed in *E. coli Rosetta2(DE3) pLysS* (Novagen) and purified by SP-Sepharose and Superdex 200 chromatography. Purified protein (5.75 mg/ml) was incubated on ice with 2 mM AMP·PNP for 2 hr prior to mixing with 50 mM NaPO_4_ pH 6.8, 7 mM DTT, 10 mM MgCl_2_, 0.7 M NaCl, 13% w/v PEG8000 for crystallization at 18 °C. Crystals appeared in four days and were harvested in cryoprotection buffer (reservoir solution containing 0.5 M NaCl + 25% glycerol) and flash-frozen in liquid nitrogen.

### X-ray diffraction data collection and processing

KIFC1 diffraction data were collected at Argonne National Laboratory (ANL) Advanced Photon Source (APS) on beamline 23ID-B (GM/CA), KIFC3 diffraction data at CHESS on beamline F1, and Ncd diffraction data at National Synchrotron Radiation Research Center (NSRRC; Hsinchu, Taiwan) on beamline BL13B1. Diffraction data were reduced with HKL2000^49^ or XDS^50^/AIMLESS^51^. Merging statistics indicated significant diffraction anisotropy for the KIFC1 and KIFC3 structures. PHASER^52^ was used for molecular replacement. The KIFC1 structure was solved using PDB 2NCD^17^ as a search model, the KIFC3 structure using PDB 1F9T as a search model, and the Ncd structure using PDB 1N6M^18^ as a search model.

A RESOLVE^53^ script was used for iterative phase modification and automated model building for KIFC1. Where electron density was not clear enough to confirm correctness of the sequence register outright, we referred to the higher resolution structure of a similar protein, PDB 1F9T^54^ for guidance. The KIFC3 model was automatically rebuilt with ARP/wARP^55^, followed by iterations of manual rebuilding and restrained refinement.

COOT^56^ was used for interactive model rebuilding for all three proteins, and REFMAC^57^ and PHENIX.REFINE^58^ were used for restrained model refinement. Model geometry and atomic displacement parameters were analyzed using MOLPROBITY^59^ and the PARVATI server^60^, respectively. Motivated in part by a PDB-REDO^61^ model of the KIFC3 structure, we undertook further refinement against reprocessed diffraction data, which also included reassignment of cross-validation flags and subsequent torsion angle simulated annealing for all three structures. PDB_EXTRACT^62^ and IOTBX^63^ were used to prepare the models for PDB deposition. The originally deposited KIFC1 (PDB 2REP) and KIFC3 (PDB 2H58) coordinates have been replaced by those for the new KIFC1 (PDB 5WDH) and KIFC3 (PDB 5WDE) models.

### Structural analysis

Structures were displayed and superposed in Chimera^64^. Dimensions of space-filled bound inhibitors were measured in Chimera. Inhibitor structures in Fig. 1C and Supplementary Fig. S2 were displayed using PubChem Sketcher v2.4. Docking of the KIFC1 inhibitor, AZ82, into the KIFC1 α4/α6 cleft was performed using PyRx^44^. Prior to docking, AZ82 (PubChem CID 91885438) was energy minimized using the Universal Force Field^65^ and several KIFC1 residues (Y499, G568-E570, S595-L601, M636, F656, A657, V660), or corresponding Ncd or KIFC3 residues, were made flexible, then docking calculations were performed using the PyRx Vina Wizard.

### Data availability statement

Diffraction data sets for KIFC1 and KIFC3 have been uploaded into the NIH Integrated Resource for Reproducibility in Macromolecular Crystallography (IRRMC) to make the original data more widely available. Coordinates for the KIFC1 (5WDH), KIFC3 (5WDE) and Ncd (5W3D) models have been deposited in the PDB for release upon publication. Other data reported here are available on request.

## Electronic supplementary material

Supplementary Information
