# Supplementary material for: Structural basis of small molecule ATPase inhibition of a human mitotic kinesin motor protein
Source: Sci Rep. 2017 Nov 9;7:15121. doi: 10.1038/s41598-017-14754-6 (PMC5680195; doi:10.1038/s41598-017-14754-6)
Supplement: Supplementary file 1 — Supplementary Information [file 41598_2017_14754_MOESM1_ESM.pdf]

Supplementary Information

Structural basis of small molecule ATPase inhibition of a human mitotic kinesin motor protein

Hee-Won Park[1,ƒ], Zhujun Ma[2,3], Haizhong Zhu[1,fl], Shimin Jiang[4,5,‡], Robert C. Robinson[4,5,6] & Sharyn A. Endow[2,3,7,*]

[1]Structural Genomics Consortium, University of Toronto, Toronto, ON M5G 1L7, CAN; [2]Neuroscience & Behavioural Disorders Programme, [3]Cancer & Stem Cell Biology Programme, Duke-National University of Singapore Medical School, SG 169857; [4]Institute of Molecular and Cell Biology, A*STAR (Agency for Science, Technology and Research), Biopolis, SG 138673; [5]Department of Biochemistry, National University of Singapore, SG 117597; [6]Research Institute for Interdisciplinary Science, Okayama University, Okayama 700-8530, JP; [7]Department of Cell Biology, Duke University Medical Center, Durham, NC 27710, USA

[ƒ]Current address: Department of Biochemistry and Molecular Biology, Tulane School of Medicine, New Orleans, LA 70112, USA

[fl]Current address: AbbVie, Inc., North Chicago, IL 60064 USA

[‡]Current address: NTU Institute of Structural Biology, Nanyang Technological University, Singapore 636921, SG

*Corresponding author

Fig S1

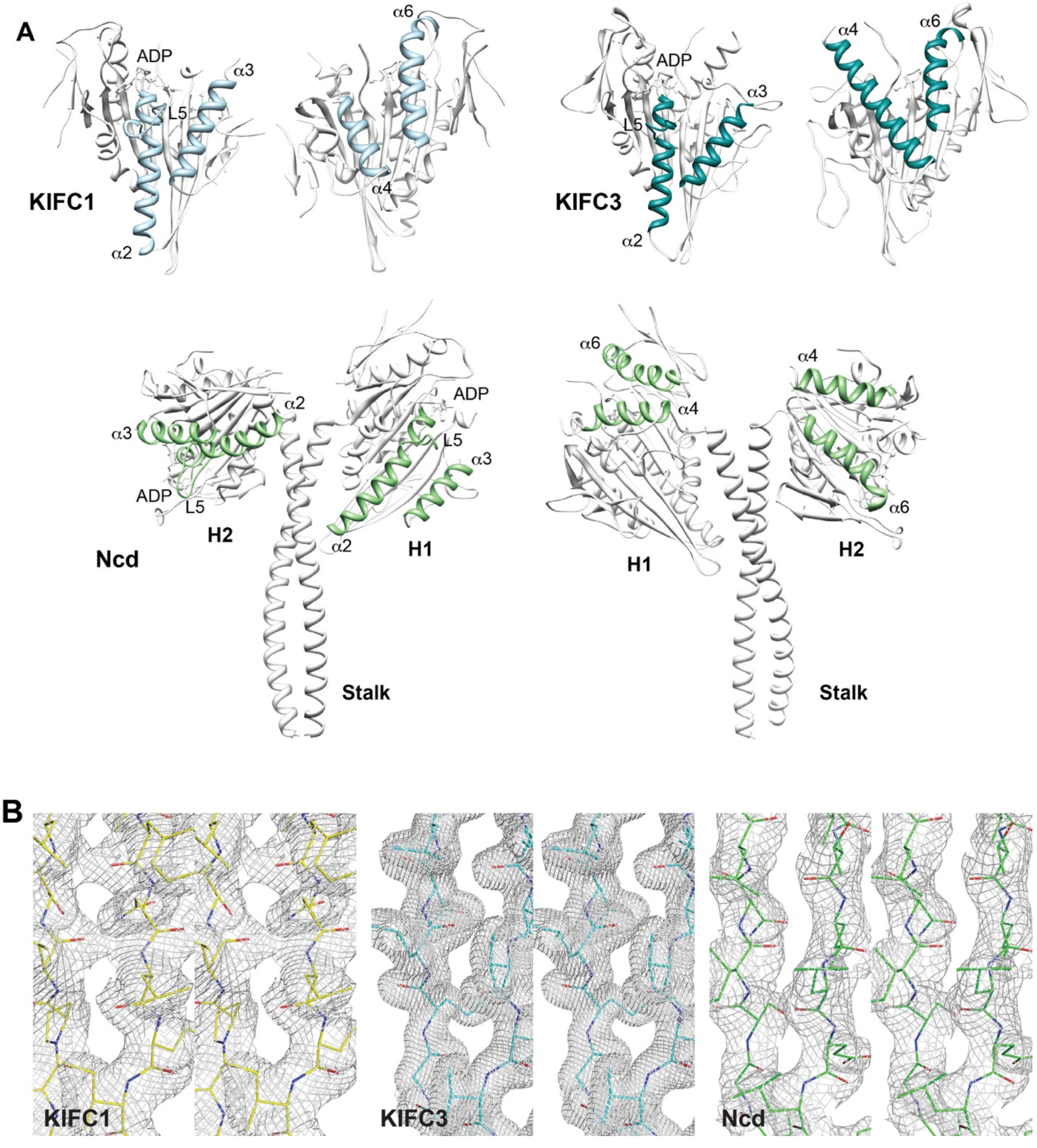

Supplementary Fig. S1 New kinesin-14 crystal structures. **A**) Top, Left, KIFC1 motor domain (PDB 5WDH). The known kinesin inhibitor-binding sites are formed by L5/α2/α3 (left) and α4/α6 (right), pale blue. Helix α2 is interrupted by loop L5 (left). The N-terminus of helix α2 that lies before L5 is not shown as part of the L5/α2/α3 inhibitor binding site in other figures, but is

shown here in all three motors for illustration. Top, Right, KIFC3 motor domain (PDB 5WDE). L5/α2/α3 (left) and α4/α6 (right), dark cyan. Bottom, Ncd dimeric motor (PDB 5W3D). The coiled-coil stalk and head H1 are rotated relative to H2. Heads H1 and H2 are thought to represent different states of the motor. L5/α2/α3 (left) and α4/α6 (right), green. **B**) Stereo images of electron density maps. 2 F_o-F_c maps of regions from strands β6 and β7, contoured at 1.0 sigma. Produced in PyMol. Left, KIFC1. Residues F542-I546, A559-L563 (carbon, yellow). Center, KIFC3. Residues L651-V655, G668-L672 (carbon, cyan). Right, Ncd. Residues T557-L561, G574-L578 (carbon, green).

Fig S2

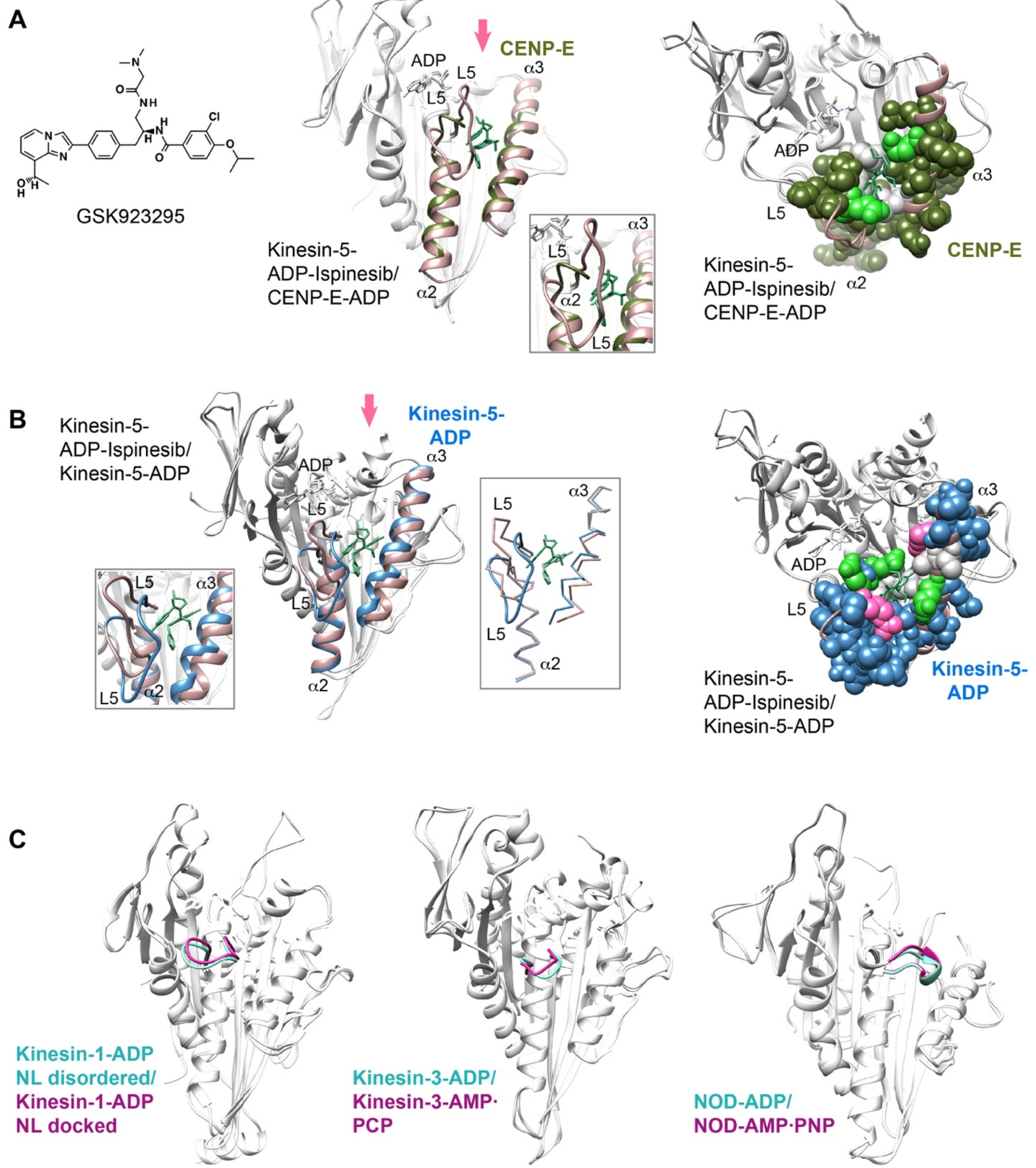

Supplementary Fig. S2 Kinesin L5/α2/α3 inhibitor binding site. **A**) Kinesin-7 CENP-E GSK923295 inhibitor. Left, GSK923295 structure. Center, CENP-E-ADP (PDB 1T5C; L5/α2/α3, olive green) superposed with kinesin-5-ADP-ispinesib (PDB 4AP0; L5/α2/α3, dark pink;

ispinesib, dark green). Inset, close-up view of binding site. Arrow, line of view for structure at right. Right, space-filled L5/α2/α3 pocket, viewed from the top as indicated by arrow (center). Hydrophobic residues, gray; acidic residues, green. ADP, white. **B**) Kinesin-5 with and without bound inhibitor. Left, kinesin-5-ADP (PDB 1II6; L5/α2/α3, blue) superposed with kinesin-5-ADP-ispinesib. Left inset, close-up view of binding site. Right inset, carbon backbone trace of the L5/α2/α3 cleft protein chains. Arrow, line of view for structure at right. Right, Space-filled L5/α2/α3 pocket, viewed from the top, as indicated by arrow (left). Hydrophobic residues, gray; basic residues, hot pink; acidic residues, green. ADP, white. Note that the N-terminus of helix α2 that lies before L5 (see Supplementary Fig. S1) is not shown as part of the L5/α2/α3 binding pocket in this or other figures. **C**) Loop L5 structure in different conformational states in other kinesins. Left, kinesin-1-ADP neck linker (NL) disordered (PDB 1BG2; L5, cyan) superposed with kinesin-1-ADP NL docked (PDB 1MKJ; L5, purple). Center, kinesin-3-ADP (PDB 1I5S; L5, cyan) superposed with kinesin-3-AMP·PCP (PDB 1I6I; L5, purple). Right, orphan kinesin NOD-ADP (PDB 3DC4; L5, cyan) superposed with NOD-AMP·PNP (PDB 3DCB; L5, purple).

The kinesin-7 CENP-E inhibitor, GSK923295, was interpreted to bind to the L5/α2/α3 pocket, based on mutant analysis and photo-affinity labeling of a structurally similar compound, GSK-1[25]. Unlike GSK923295, GSK-1 binding is competitive with ATP[25]. GSK-1 was later reported to be selective for kinesin-5 and to bind to a second kinesin inhibitor binding site, the α4/α6 site[41]. From the mutant analysis, GSK923295 probably binds to the kinesin-7 CENP-E L5/α2/α3 site, whereas GSK-1 most likely binds to the kinesin-5 α4/α6 cleft.

Fig S3

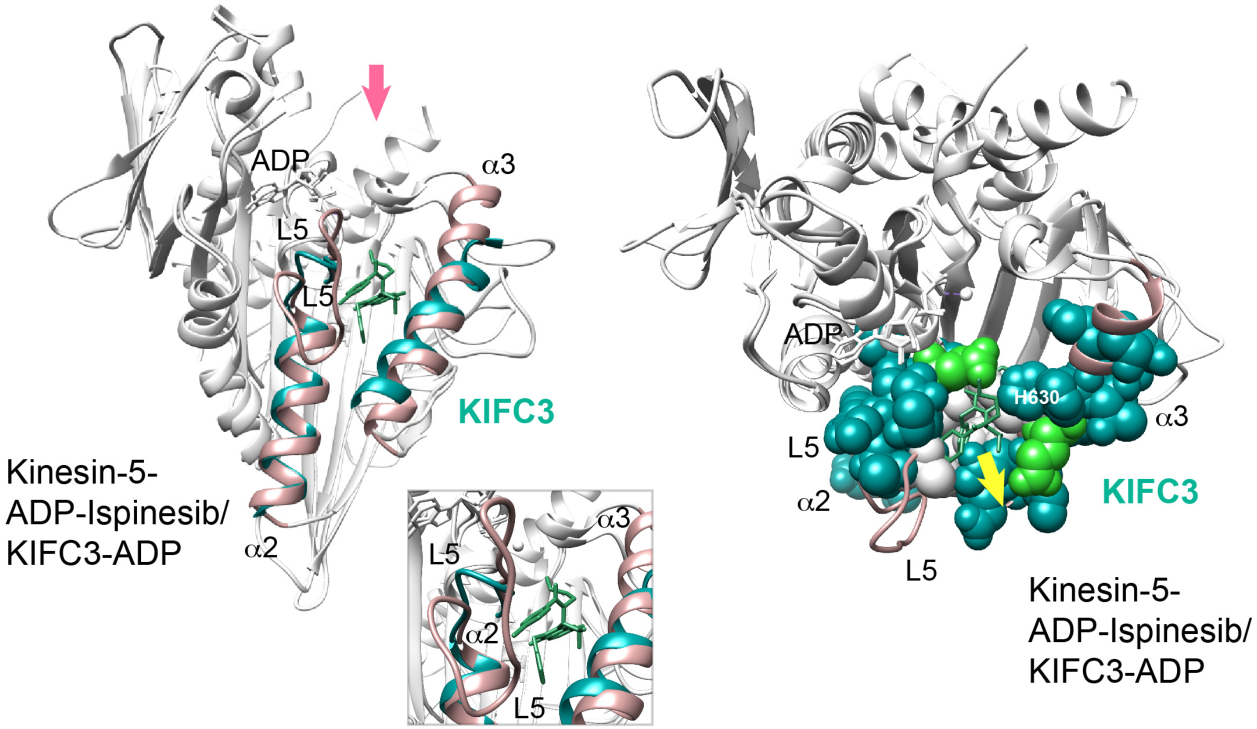

Supplementary Fig. S3 Kinesin-14 KIFC3 L5/α2/α3 inhibitor binding site. Left, KIFC3-ADP (PDB 5WDE; L5/α2/α3, dark cyan) superposed with kinesin-5-ADP-ispinesib (PDB 4AP0; L5/α2/α3, dark pink; ispinesib, dark green). Inset, close-up view of binding site. Arrow, line of view for structure at right. Right, space-filled L5/α2/α3 pocket, viewed from the top, as indicated by arrow (hot pink, left). Hydrophobic residues, gray; acidic residues, green. ADP, white. Arrow (yellow), opening at bottom of pocket.

Fig S4

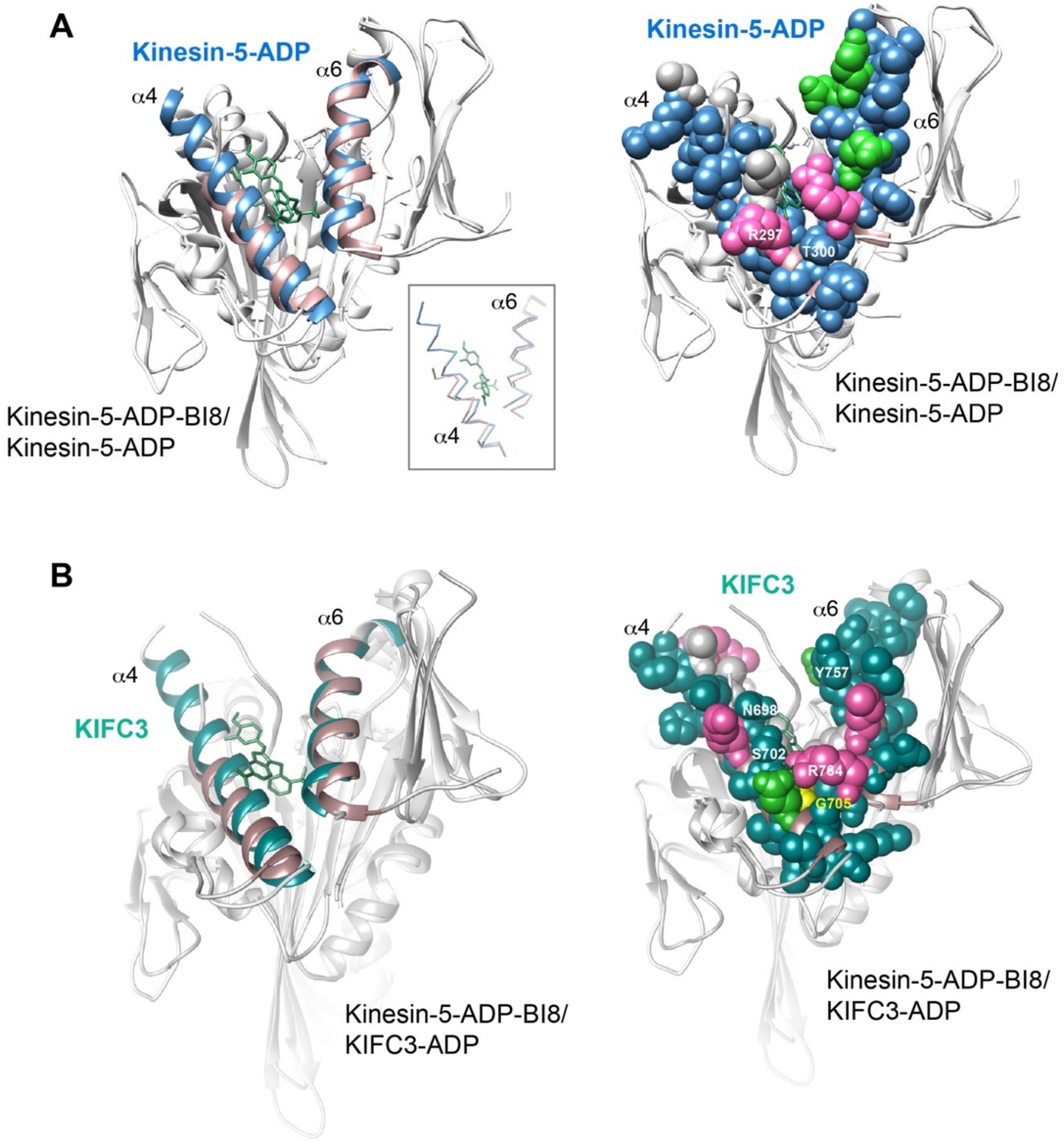

Supplementary Fig. S4 Kinesin $\alpha4/\alpha6$ inhibitor binding site. **A**) Kinesin-5 with and without bound inhibitor. Left, kinesin-5-ADP (PDB 1II6; $\alpha4/\alpha6$, blue) superposed with kinesin-5-ADP-BI8 (PDB 3ZCW; $\alpha4/\alpha6$, dark pink; BI8, dark green). Inset, carbon backbone trace of the $\alpha4/\alpha6$ cleft protein chains. Right, Space-filled $\alpha4/\alpha6$ pocket. Hydrophobic residues, gray; basic residues, hot pink; acidic residues, green. **B**) Kinesin-14 KIFC3. Left, KIFC3-ADP (PDB 5WDE; $\alpha4/\alpha6$, dark cyan) superposed with kinesin-5-ADP-ispinesib (PDB 4AP0). Right, space-filled $\alpha4/\alpha6$ pocket. Hydrophobic residues, gray; basic residues, hot pink; acidic residues, green. KIFC3 G705, yellow, is conserved with kinesin-5 G296 (see Fig. 3).

Fig S5

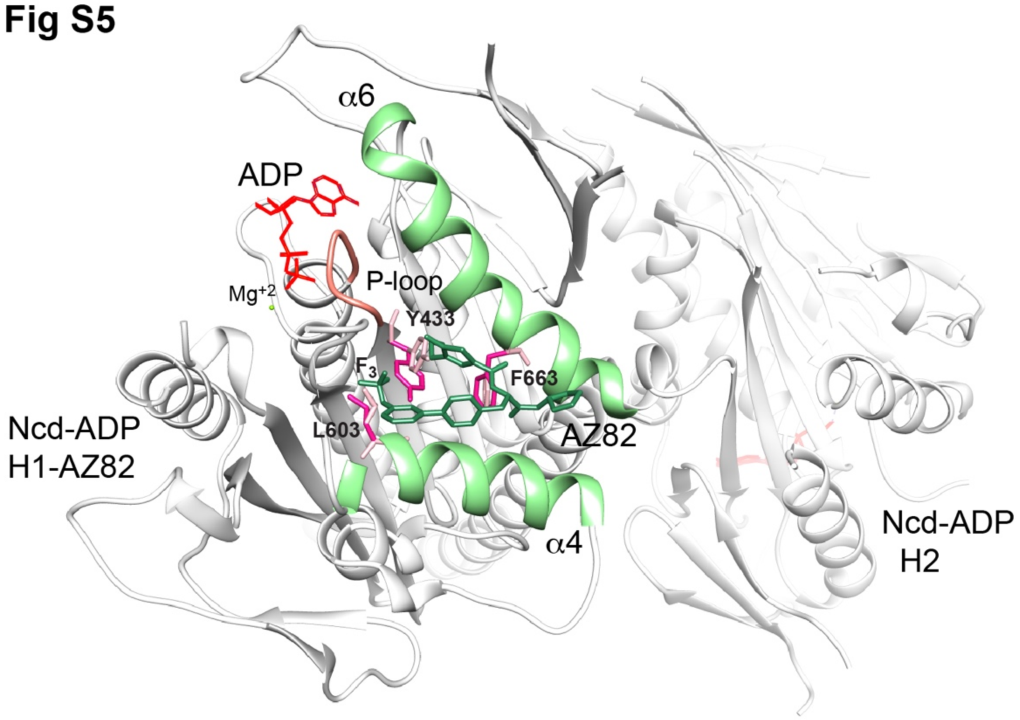

Supplementary Fig. S5 AZ82 docked into Ncd (PDB 5W3D) head H1 α4/α6 cleft. The predicted free energy change for Ncd H1 (ΔG=-7.9 kcal/mol) was close to that for AZ82 docking into the KIFC1 α4/α6 pocket (ΔG=-8.1 kcal/mol). However, AZ82 was bound to Ncd H1 with a higher predicted free energy change than head H2 (ΔG=-6.5 kcal/mol), the head positioned to bind to the microtubule – this indicates that binding by the drug is unlikely to specifically inhibit Ncd-MT activity, as observed for KIFC1. In addition, AZ82 is bound to a shallow groove in Ncd H1 and H2, as well as KIFC3, rather than with the F_3 group buried in the pocket, as observed for KIFC1 (Fig. 5), and may not be stably bound. Ncd H1 residues Y433, L603 and F663 are shown as stick models before (pale pink) and after (dark pink) docking. AZ82, dark green; Ncd H1 helices α4 and α6, light green; P-loop, coral; ADP, red; Mg^{+2}, yellow green.
